# Gene x environment interaction analysis confirms genetic modifier effects on steroid efficacy via TGF-β pathway in Duchenne muscular dystrophy

**DOI:** 10.1038/s41431-026-02110-0

**Published:** 2026-04-20

**Authors:** Veronica J. Vieland, Sang-Cheol Seok, Megan A. Waldrop, Leigh M. Gabel, Diane M. Dunn, Kevin M. Flanigan, Robert B. Weiss

**Affiliations:** 1 Department of Pediatrics (Emerita), The Ohio State University, Columbus, OH USA; 2 Department of Statistics (Emerita), The Ohio State University, Columbus, OH USA; 3 Mathematical Medicine LLC, Chicago, IL USA; 4 Center for Gene Therapy, The Abigail Wexner Research Institute at Nationwide Children’s Hospital, Columbus, OH USA; 5 Department of Pediatrics, The Ohio State University, Columbus, OH USA; 6 Department of Neurology, The Ohio State University, Columbus, OH USA; 7 Department of Human Genetics, University of Utah, Salt Lake City, UT USA

**Keywords:** Genetics research, Genetic association study

## Abstract

This paper continues our development of methods for discovery of genetic modifiers of the Duchenne muscular dystrophy (DMD) phenotype. DMD is an X-linked recessive disorder involving progressive muscle tissue loss with replacement by fat and fibrotic tissue, leading in most cases to loss of ambulation (LOA) by early to mid-adolescence. The standard pharmacologic treatment is corticosteroid administration, which increases average LOA by 2–3 years. There is variation in LOA due to specific *DMD* mutations, some of which permit the production of residual or partial dystrophin protein and lead to milder phenotypes. But there is also believed to be variation due to genetic modifiers acting even in patients whose *DMD* mutations preclude dystrophin production altogether, based in part on animal models, and several genes have been implicated as potential modifiers of LOA in DMD patients. Here we consider whether the mechanism of action of any of these genes might be to influence LOA by modifying the effects of corticosteroid exposure. We develop and evaluate a novel statistic, the PPI_GxE_; we consider the issue of potential “phenocopies,” or individuals whose late LOA might be due to residual dystrophin production; and we apply our approach to 12 candidate SNPs using our DMD dataset. We find evidence of genotype x steroid interaction effects for 4 out of the 12 SNPs we tested, which can be linked to the TGF-β pathway. These results corroborate the hypothesis that modifiers in the TGF-β pathway affect LOA by modulating the efficacy of corticosteroid administration.

## Introduction

This paper continues work on the discovery and understanding of genes that can modify the Duchenne muscular dystrophy (DMD) phenotype. DMD is an X-linked recessive disorder affecting $\approx$ 1 in 5000 live male births [1, 2], involving progressive muscle tissue loss with replacement by fat and fibrotic tissue. Patients typically become reliant on wheelchairs by early to mid-adolescence, but some maintain ambulation substantially longer, and age at loss of ambulation (LOA) is an important clinical indicator of disease progression. DMD is currently without a cure. Corticosteroid (steroid) administration is a common treatment, increasing average LOA by 2–3 years.

The primary determinant of LOA among DMD patients is the specific mutation that a patient carries, with some mutations permitting residual or partial dystrophin production, which can ameliorate the phenotype [3, 4]. But even among patients whose mutations preclude the production of any dystrophin there is variability in LOA, and modifier genes have been shown to influence the rate of disease progression in animal models [5, 6]. The discovery of DMD modifier genes in humans would have implications both for therapeutics and for the design of DMD clinical trials, and is therefore of clinical interest.

In humans, previous studies have implicated the genes *SPP1*, *LTBP4*, *THBS1*, *CD40*, *ACTN3*, and *TCTEX1D1* [7–10]. Most recently, we performed a genome wide association study (GWAS) in the largest sample assembled to date [11] (*N* = 419 DMD cases), comprising data from the United Dystrophinopathy Project (UDP), a multisite consortium [12–14]. One distinguishing feature of our study was the use of stringent subject inclusion criteria based on *DMD* mutation type, aimed at excluding individuals with any potential for residual dystrophin expression, including those with mutations known to permit only minimal or trace amounts. In addition, we used statistical methods specifically adapted to our application [15, 16] and developed an unbiased in silico pipeline to infer functional relationships between SNPs of interest and specific genes. The result was discovery of compelling evidence for association with LOA for multiple SNPs, implicating the genes *ETAA1*, *NCALD*, *GALNTL6*, *MAN1A1*, *ADAMTS19*, and *PARD6G1* [11]. Follow-up using a zebrafish DMD model appears to have confirmed a role for 4 of these genes (etaa1a/etaa1b, galntl6, man1a1, adamts19) in modifying the DMD phenotype [17].

One mechanism by which any given modifier gene might affect LOA could be through interaction with steroid exposure, and this is the primary focus of the current paper. As mentioned, our previous work has relied on specially tailored statistical methods, which we have shown to have distinct advantages over standard methods such as Cox proportional hazards (CPH) modeling for our application [16]. These advantages included better operational characteristics in the modest sample sizes available for the study of DMD; the ability to determine evidence either for or against an effect; and the ability to interrogate effects on means and/or variances. Since these features would be similarly beneficial in modeling gene by environment (GxE) interactions, here we extend our statistical methodology to cover this case, describing our new interaction statistic, which we call PPI_G×E_ (Posterior Probability of GxE Interaction), and evaluating its behavior using simulations.

The search for genetic modifiers of LOA in DMD patients is complicated by the possibility that individuals with unusually late LOA might in fact be producing some dystrophin. Despite stringent exclusion criteria, designed to eliminate leaky mutations from the data set, it remains possible that some late LOA individuals might in fact be carrying such mutations. The simulations described below created an opportunity to revisit the issue of how to handle late LOA values. This is a secondary focus of the current paper. Finally, taking what we learned from these investigations, we apply our new statistic to the UDP data, finding evidence of genotype x steroid interaction for *NCALD* (or possibly *KLF10*, see below), *LTBP4, THBS1* and *CD40*.

## Materials and methods

In this section, we describe (1) the statistical methods, (2) the simulations procedures used in evaluating the methods, and (3) the DMD data set to which these methods are then applied.

### Statistical methods

PPI_G×E_ is a function of the likelihood ratio (LR) comparing two hypotheses, H_1_ and H_2_. Both hypotheses allow that a binary environmental exposure affects the phenotype of interest, and also that genotypes at the particular SNP being evaluated affect the phenotype (or at least, that they show statistical evidence of an effect). Specifically, here we assume that both steroid exposure and the SNP under consideration have an effect on LOA.

Under H_1_, the effects of steroid exposure and genotype are assumed to be independent of one another, that is, the impact of steroids is assumed to be the same regardless of SNP genotype. Under H_2_, the impact of steroid exposure is allowed to vary with SNP genotype. Let the genotypes be represented by 11, 12, and 22 (where “1” is the minor allele). Let the genotypic mean and standard deviation (s.d.) of LOA be ${\mu }_{i,j},\,{\sigma }_{i,j}$ for the *i*^*th*^ steroid group (N, Y) and the *j*^*th*^ genotype (11, 12, or 22). Under H_1_, regardless of genotype the mean LOA in the steroid Y group is obtained by adding a term $\alpha$ to the mean LOA in the steroid N group; similarly, we represent the change in the s.d. from the steroid N to steroid Y group by a single factor $\beta$. By contrast, under H_2_ each genotype is permitted its own values of $\alpha$ and $\beta$. Table 1 shows the parameterization of the two hypotheses. The hypotheses can be succinctly represented as H_1_: ${\alpha }_{11}={\alpha }_{12}={\alpha }_{22}$, ${\beta }_{11}={\beta }_{12}={\beta }_{22}$ vs. H_2_: ${\alpha }_{11}\ne {\alpha }_{12}\ne {\alpha }_{22}$, ${\beta }_{11}\ne {\beta }_{12}\ne {\beta }_{22}$, where “$\ne$ means only that any of the equalities need not hold. This gives us ${{LR}}_{G\times E}=\frac{P\left({data}|{H}_{2}\right)}{P\left({data}|{H}_{1}\right)}=\frac{{P}_{{H}_{2}}\left({Data}|{\alpha }_{11},{\alpha }_{12},{\alpha }_{22},\,{\beta }_{11},\,{\beta }_{12},\,{\beta }_{22}\right)}{{P}_{{H}_{1}}\left({Data}|\alpha ,\beta \right)}=\frac{{\prod }_{i=1}^{3}{\prod }_{{x}_{j}\in i}\Phi ({x}_{j};\,{{\mu }_{i}+\alpha }_{i},\,{{\,\sigma }_{i}\times \beta }_{i})}{{\prod }_{i=1}^{3}{\prod }_{{x}_{j}\in i}\Phi ({x}_{j};\,{\mu }_{i}+\alpha ,\,{\sigma }_{i}\times \beta )}$, where *i* ranges over genotypes; *j* ranges over individuals with a given genotype; ${\mu }_{i},\,{\sigma }_{i}$ are the mean, standard deviation for the *i*^*th*^ genotype in the Steroid = N group, and $\Phi$ is the normal probability density function. Note that we assume normality of LOA at the genotypic level but not at the population level.

**Table 1:** Hypotheses and notation.

Genotype	11	12	22
**H**_**1**_			
steroid N	${\mu }_{11,N}$, ${\sigma }_{11,N}$	${\mu }_{12,N}$, ${\sigma }_{12,N}$	${\mu }_{22,N,}{\,\sigma }_{22,N}$
steroid Y	${\mu }_{11,N}+\alpha$, ${\sigma }_{11,N}\times \beta$	${\mu }_{12,N}+\alpha$, ${\sigma }_{12,N}\times \beta$	${\mu }_{22,N}+\alpha$, ${\sigma }_{22,N}\times \beta$
**H**_**2**_			
steroid N	${\mu }_{11,N}$, ${\sigma }_{11,N}$	${\mu }_{12,N}$, ${\sigma }_{12,N}$	${\mu }_{22,N}$, ${\sigma }_{22,N}$
steroid Y	${\mu }_{11,N}+{\alpha }_{11}$, ${\sigma }_{11,N}\times {\beta }_{11}$	${\mu }_{12,N}+{\alpha }_{12}$, ${\sigma }_{12,N}\times {\beta }_{12}$	${\mu }_{22,N}+{\alpha }_{22}$, ${\sigma }_{22,N}\times {\beta }_{22}$

Continuing the development of the statistical framework developed for our GWAS analysis [18], we define the Bayes ratio (BR) as

$${{{\rm{BR}}}}_{{{\rm{G}}}\times {{\rm{E}}}}={\log }_{10}\int {{LR}}_{{GxE}}\left({{\boldsymbol{\gamma }}}\right)f\left({{\boldsymbol{\gamma }}}\right)d{{\boldsymbol{\gamma }}}$$

where the single integral stands in for multiple integration over the vector ${{\boldsymbol{\gamma }}}=({\alpha }_{11},\,{\alpha }_{12},\,{\alpha }_{22},{\beta }_{11},\,{\beta }_{12},{\beta }_{22})$. See Supplemental Material for additional details.

In order to maintain comparability of scale with our primary association statistic, the TE-PPLD (Time to Event Posterior Probability of (trait-marker) Linkage Disequilibrium), we convert the BR onto the posterior probability scale via Bayes’ theorem:

$${{PPI}}_{{GxE}}=\frac{{\pi \times 10}^{{{BR}}_{{GxE}}}}{{\pi \times 10}^{{{BR}}_{{GxE}}}+(1-\pi )}$$

where $\pi$ is set to 0.0004, the same point-prior used in calculating the PPLD. Since the true prior probability of an interaction is unknowable, there is no empirical basis for preferring any value over another; this particular choice for $\pi$ is one of convenience only, allowing us to maintain the same heuristics for evaluating evidence strength that we developed for the PPLD. See also Supplemental Material for further discussion.

${{{\rm{PPI}}}}_{{{\rm{G}}}\times {{\rm{E}}}}$ calculations were done in MATLAB (2021.9.10.0.1739362 (R2021a)). Code is available at https://github.com/MathematicalMedicine/DMD/.

### Simulation methods

In order to assess performance characteristics of PPI_G×E_ we simulated data under conditions designed to mimic features of our DMD data set using previously described methods [16]. In brief, our base model uses a sample size of *N* = 400 unrelated individuals, split 30%, 70% into two covariate levels (*y* = 1, 2, respectively) to mimic our DMD dataset. SNP minor allele frequencies (MAFs) were randomly drawn from an empirical distribution function based on the MAFs from our real genome scan (mean =  0.23, median = 0.20, s.d. = 0.14), and individuals were randomly assigned a genotype for a 2-allele locus (with alleles 1, 2) as a function of the MAF assuming Hardy Weinberg equilibrium. We simulated 1000 replicates of 400 individuals under each of 25 different models, chosen to illustrate what we thought in advance might represent a range of plausible effect types and sizes. Each model is specified by 6 parameters: 3 genotypic means and 3 genotypic standard deviations. Genotypic effects in the steroid = N group are specified as recessive (REC), additive (ADD) or dominant, (DOM) with varying levels of effect size; the effects of steroid are varied with respect to whether or not there is a genotype x steroid interaction, and if there is, with respect to the size and pattern (REC, ADD, DOM or over-dominant (OTHER)) of the steroid effect, with interaction effects on means only (M), on standard deviations only (S), or on both means and standard deviations (MS). Details are given in Supplemental Material.

### The UDP data set

Below we analyze data from the UDP, the same data set used in our previous GWAS as described in [11]. In brief, LOA was defined by full-time wheelchair use (requiring wheelchair within the home) and recorded to the nearest month when available, and otherwise to the nearest half-year of age. Glucocorticoid treatment (Steroid Y) was defined as use of any steroid regimen for more than 6 months that began at least 6 months prior to loss of ambulation. Untreated (Steroid N) was defined as never exposed, exposed to any steroid regimen for less than 6 months, or onset of treatment less than 6 months prior to loss of ambulation. Genotyping methods and cleaning protocols are also described in [11].

The full sample size is *N* = 419. As mentioned above, our intent was to include only subjects with true loss-of-function mutations within *DMD*, excluding genotypes known to be associated with milder disease severity such as deletion of exons 3–7, deletion of exon 45, or nonsense mutations within the exon 23–42 region [13]. Based on available knowledge at that time, we included individuals with duplications of exon 2 (Dup2) mutations. Subsequently, it was shown that some Dup2 carriers display milder phenotypes [19]. There are 12 such individuals in the UDP dataset, 2 of whom have LOA ≥ 20. Here, we analyze the data both including and excluding these individuals (see also [11]). We apply the PPI_GxE_ to the 6 SNPs with primary association signal TE-PPLD > 0.40 from our previous genome scan as well as to the 6 candidate SNPs from previous studies also considered in that paper [11].

## Results

In this section we (1) show the power of the PPI_GxE_ based on the simulated models, (2) focus specifically on the issue of whether to include individuals with late LOA, and (3) apply the PPI_GxE_ to the UDP data set.

### Power of the PPI_GxE_

Table 2 shows power to exceed the conventional thresholds for weak, moderate or strong evidence (0.04, 0.10, and 0.40, respectively) adopted in [11]. We also show the probability of obtaining evidence against interaction (PPI < 0.0004). At a sample size of *N* = 400, power to detect strong evidence is low to middling in most cases. In general, models with larger interaction effect sizes show higher power, as expected, as do models with interaction effects on both means and standard deviations (e.g., models A18–A20). However, with the exception of models A21-A23 (the deleterious models), the probability of exceeding the given thresholds for interaction is notably higher for all of the alternative models than for either of the null models, although it is also notable that the probability of obtaining evidence against interaction can be quite high even in models with a true interaction effect. This reflects the fact that our current sample size is simply not large enough to ensure that we will be able to distinguish evidence against from evidence for interaction in the presence of real interactions. (With increased sample size both power to correctly find evidence for interaction and also power to correctly find evidence against interaction will increase.) We note that power to detect deleterious effects of the rare allele are particularly low, which is not surprising given the extreme skewness of the LOA distribution; there simply is not a lot of room between the minimum observed age of 6 and the average age at LOA for detectable effects given our sample size.

**Table 2:** Proportion of replicates (out of 1000/model) achieving PPI_GxE_ < or $\ge$ thresholds as shown.

		PPI_GxE_ threshold			
Model	Model	<0.0004	≥0.04	≥0.1	≥0.4
N1	Steroid Only	93.5	1.9	1.6	1.2
N2	No Interaction	95.9	1.1	0.8	0.6
A1	AddAddM1	49.4	30.4	27.7	22.6
A2	AddAddM2	29.6	51.8	48.5	42.1
A3	AddAddM3	50.8	29.4	26.1	19.4
A4	AddAddM4	34.5	44.6	41.6	34.9
A5	AddAddS1	62.7	22.3	20.4	16.3
A6	AddAddS2	46.2	35.8	31.9	27.4
A7	AddAddS3	33.8	45.0	40.6	34.5
A8	AddAddMS1	55.9	27.2	24.5	19.8
A9	AddAddMS2	52.8	28.7	25.0	19.4
A10	AddAddMS3	41.0	39.2	36.3	30.0
A11	AddAddMS4	47.7	31.1	28.5	23.0
A12	RecRecMS1	60.3	28.1	27.4	25.0
A13	RecRecMS2	59.1	32.2	30.4	28.1
A14	DomDomMS1	24.6	59.4	55.0	46.9
A15	DomDomMS2	21.6	64.1	60.8	54.5
A16	RecOtherS	14.3	72.9	71.0	64.5
A17	DomOtherS	17.1	67.0	64.0	56.7
A18	RecOtherMS	11.6	78.4	75.7	69.9
A19	DomOtherMS1	10.4	78.9	76.1	70.4
A20	DomOtherMS2	5.1	86.6	84.4	80.9
A21	DeleteriousM1	96.3	1.4	1.3	0.4
A22	DeleteriousM2	88.6	3.9	2.5	1.9
A23	DeleteriousM3	73.5	11.3	8.7	6.5

**Fig. 1:**
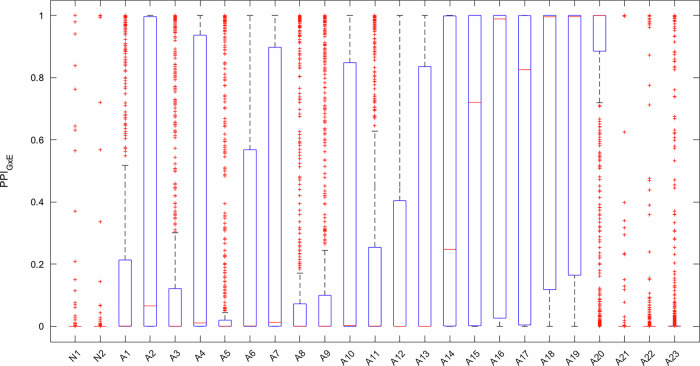
Boxplots showing distribution of PPI_GxE_ for each simulated generating model. Each column represents the distribution of PPI_GxE_ across 1000 replicates for one generating model. For Models N1, N2, A21, A22, and A23, the proportion of replicates with PPI_GxE_ < 0.0004 (that is, giving evidence against interaction) is 93.5%, 95.9%, 96.3%, 88.6%, and 73.5%, respectively. Note that when the great majority of results are very close to 0, the 25–75% interquartile range (represented by a box) becomes indistinguishable from 0, so that only “outliers” are visible in the plot.

Two other features of these simulations are worth noting. First, there is considerable variability in results from replicate to replicate, as shown in Fig. 1. This is not an artifact of the particular test statistic being used; for example, we have previously illustrated high levels of variability under similar simulation conditions for *p*-values from CPH modeling [16]. Rather, this level of variability is a feature of the simulation set up, in which there are a number of sources of variability in the underlying data (randomized genotypes, randomized phenotypes conditioned on genotype and covariate status, randomized observation times). We return to this point in the Discussion.

Second, overall the distribution of LOA in our generating models mimics what we see in our DMD data set fairly well (Table 3), as intended; the row labeled “UDP” represents the DMD data set, while the remainder represent the various simulated models. One noteworthy feature of the simulations is the occurrence of substantial numbers of individuals with LOA ≥ 20.

**Table 3:** Key characteristics of simulated data.

		LOA		# with LOA $\ge$ 20		
Model		mean	s.d.	mean	std	max
UDP	Real DMD Data	12.1	3.1	10 ind’s with LOA ${{\boldsymbol{\ge }}}$20		
N1	Steroid Only	12.0	3.5	6.8	2.8	22
N2	No Interaction	12.0	3.5	7.4	2.9	18
A1	AddAddM1	12.2	3.1	3.3	3.3	19
A2	AddAddM2	12.4	3.2	4.8	4.4	21
A3	AddAddM3	11.8	3.2	4.0	3.8	19
A4	AddAddM4	11.9	3.4	5.7	5.5	29
A5	AddAddS1	11.8	3.0	4.2	3.8	23
A6	AddAddS2	11.9	3.3	6.9	5.4	33
A7	AddAddS3	11.6	3.6	10.6	7.7	37
A8	AddAddMS1	12.0	3.2	5.5	4.7	29
A9	AddAddMS2	12.2	3.2	5.3	4.4	24
A10	AddAddMS3	12.2	3.3	7.5	6.2	37
A11	AddAddMS4	11.7	3.4	8.0	6.9	34
A12	RecRecMS1	11.5	2.8	2.5	2.9	15
A13	RecRecMS2	11.5	2.9	3.2	3.7	19
A14	DomDomMS1	12.5	3.8	15.7	11.6	56
A15	DomDomMS2	12.8	4.0	19.9	14.5	74
A16	RecOtherS	11.8	3.3	7.3	4.6	24
A17	DomOtherS	12.2	3.5	10.0	6.0	34
A18	RecOtherMS	12.8	3.5	9.5	6.8	34
A19	DomOtherMS1	12.8	3.9	18.1	12.2	62
A20	DomOtherMS2	12.9	4.1	20.8	13.6	66
A21	DeleteriousM1	12.5	2.7	1.4	1.2	7
A22	DeleteriiousM2	12.4	2.7	1.3	1.2	6
A23	DeleteriousM3	12.2	2.7	1.2	1.1	7

### Sensitivity to outlier LOA values

In order to evaluate sensitivity of the PPI_GxE_ to outlier LOA values, we focused one of the alternative models (A18) along with the two “null” models. We chose model A18 because (i) it has mean (s.d.) LOA very close to the UDP data set; (ii) it has mean number of individuals with LOA ≥ 20 very close to the actual number is the UDP data set; and (iii) it yields reasonably high power (Table 2), which permits us to observe both positive and negative effects of changes in the data. We reanalyzed the 1000 A18 replicates in 3 different ways: (i) DROP_LOA: for each replicate, we dropped all individuals with LOA ≥ 20; (ii) DROP_rand: for each replicate, we dropped the same number of individuals as in (i), however, we dropped individuals at random rather than based on LOA; (iii) REPLACE: we replaced the genotypes of those same high LOA individuals with randomly generated genotypes, based on the MAF for the given replicate and without consideration of genotype, but retaining the original LOA value. Comparing (i) and (ii) allows us to see the extent to which effects of dropping high LOA individuals are simply an artifact of reducing the sample size; while (iii) mimics the situation in which the high LOA individuals are in fact a distinct group whose late LOA is due, e.g., to their primary *DMD* mutation and not to the effects of the SNP being assayed. In what follows, for brevity we refer to these individuals as “phenocopies.”

**Fig. 2:**
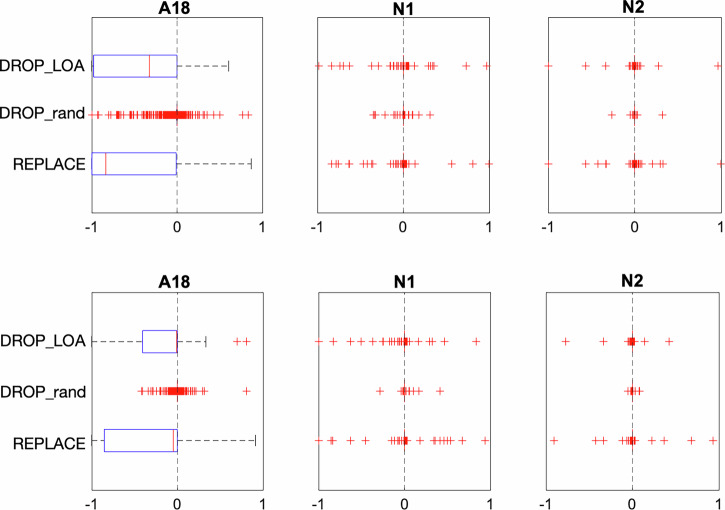
Effects on PPI_GxE_ of modifying data sets using various criteria, for generating models A18 (alternative), N1 and N2 (null). The first row shows results of altering the dataset based on all individuals (per replicate) with LOA ≥ 20, as described in the text; the second row shows results of altering the dataset based on just two high-LOA individuals. Within subplots, the three rows (DROP_LOA, DROP_RAND and REPLACE) each represent the new PPI_GXE_, after altering the data set, minus the original PPI_GXE_, prior to altering the data set. Because PPI_GXE_ is on the (0,..,1) scale, these differences are bounded by –1, 1, with 0 representing no change in result from one analysis to the other. Note that when the great majority of results are very close to 0, the box representing the 25–75% interquartile range becomes indistinguishable from 0, so that only “outliers” are visible in the plot.

Figure 2 shows the results. We can see (Fig. 2 first row) that for model A18, dropping individuals with high LOA has an almost uniformly negative impact on the PPI_GxE_, and in a large proportion of replicates this impact is severe, causing very high PPI_GxE_ to plummet. By contrast, dropping the same number of individuals at random, for the same per-replicate reduction in sample size, tends to have only a minor impact on PPI_GxE_, with both increases and decreases in scores; although even in this case there are some replicates where the random change in sample size itself has a larger impact on the results. Finally, replacing the high LOA individuals with phenocopies results in a distribution of changes in PPI_GxE_ that looks very much like what we see when we simply drop the high LOA individuals. The general trends here are unsurprising. However, these simulations by design mimic the particular situation we face in studying DMD, with the sample size, the distribution of LOA (including the number of outliers) and our inability to determine distinct underlying causes of high LOA all reflecting conditions we face in analyzing real data. What is interesting, therefore, is the extent to which decisions regarding small changes to inclusion/exclusion criteria based on LOA itself can have large impacts on results in the DMD setting.

In fact, these same patterns repeat themselves if we focus on just the 2 highest LOAs per replicate (Fig. 2 second row). This illustrates clearly that small numbers of individuals (in this case, just 2) can be highly influential; and also, that when an interaction signal disappears after dropping 2 influential individuals, this does *not* imply that the initial result was a false positive. On the contrary, we expect this drop in score to happen, particularly under the alternative hypothesis.

By contrast, under either null hypothesis, the vast majority of replicates show only very small changes in PPI_GXE_ as a result of alterations to the dataset, and those changes that we do see tend to be more randomly distributed between the negative and positive portions of the x-axis, in both rows of the figure. Although here as well large changes do occur: roughly half the time these changes are beneficial, substantially reducing initial signals that were in fact false positives; but also in a small number of replicates causing a (correctly) small PPI_GXE_ to become (spuriously) large.

To summarize: (i) under the null hypotheses, the changes to the data we have explored generally result in small and essentially random changes in the results; (ii) under the alternative hypothesis (model A18), *dropping* even a small number of individuals based on outlier LOA values tends to negatively affect results, sometimes dramatically so, when those individuals are not phenocopies; but also (iii), under the alternative hypothesis *including* individuals with outlier LOA values tends to negatively affect results, sometimes dramatically so, when those individuals are in effect phenocopies with respect to the modifier effect of the SNP being assayed. Thus, there can be high costs to either including or excluding outlier LOA individuals, depending on whether those individuals are phenocopies or not, something that we cannot know in advance in practice. Therefore, in what follows we analyze the UDP data both including and excluding Dup2 individuals, not based directly on their observed LOA, but rather on the fact that as a class they tend to have milder phenotypes, including delayed LOA [19].

### Application to UDP data set

Table 4 shows results both in the full (*N* = 419) dataset and in the reduced (no Dup2s, *N* = 407) dataset. Original association statistics (TE-PPLD) are repeated from [11] for reference purposes.

**Table 4:** Results for UDP data set.

		*N* = 419		*N* = 407	
Marker	Gene^a^	TE-PPLD	PPI_GxE_	TE-PPLD^b^	PPI_GxE_
**rs34263553**	*ETAA1*	0.87	0.0005	0.79	0.00
**rs72681143**	*NCALD*	0.77	0	0.05	**1.00**
**rs1358596**	*GALNTL6*	0.42	0	0.54	0.00
**rs10499096**	*MAN1A1*	0.62	0	0.73	0.00
**rs10077875**	*ADAMTS19*	0.44	0	0.03	0.00
**rs2061566**	*PARD6G*	0.40	0.0001	0.22	0.00
**rs28357094**	*SPP1*	0.0003	0	0.0007	0.00
**rs710160**	*LTBP4*	0.0002	**1.0**	0.0003	0.01
**rs2725797**	*THBS1*	0.0001	**1.0**	0.0001	0.00
**rs1883832**	*CD40*	0.0002	0	0.0002	**0.54**
**rs1815739**	*ACTN3*	0.0001	0	0.0002	0.00
**kgp9098261**	*TCTEX1D1*	0.0004	0	0.0001	0.00

^a^We have listed the gene we deemed most likely to be implicated based on in silico analysis; see [11] for details.

^b^Repeating the original association genome scan [11] after dropping Dup2 individuals yielded no new SNPs with PPLD ≥ 40%.

Of the SNPs originally showing strong evidence of association, one (which we previously associated with *NCALD*) shows very high (rounded to 1) evidence of an interaction with steroid exposure. This occurs only when we drop the Dup2 individuals, suggesting that for purposes of evaluating the interaction these individuals may be phenocopies. However, when we drop the Dup2 individuals, the original association signal drops from 0.77 to 0.05. The latter is still well above the prior (0.0004), and this may simply represent sampling variability acting on a true signal. The remaining SNPs with large PPI_GxE_ all come from the list of previously reported candidate genes, none of which showed evidence of association in our previous genome scan. In two cases (LTPB4 and THBS1) we have very large signals when including the Dup2 individuals, which drop off considerably but remain well above the prior in the reduced sample. In contrast, we pick up the signal in CD40 only after dropping the Dup2 individuals. Maximum likelihood estimates of the model parameters for these 4 SNPs are included in Supplemental Material.

## Discussion

We have developed a new statistic, the PPI_G×E_, for assessing evidence for or against gene x environment interaction, and evaluated the performance of this statistic using simulations designed to mimic our intended application to DMD. At the current DMD sample size of *N* = 419, and adopting even our moderate threshold for weak evidence (PPI_G×E_ $\ge$ 0.04), the PPI_G×E_ tended to have low to modest power for many of our generating models. Nonetheless, scores this high were far more likely to occur under even the weakest of our generating models than under the null hypothesis. And of course, the list of generating models we considered is by no means comprehensive, or necessarily reflective of real biological effects at play in modification of the DMD phenotype.

One potential concern is our application of the PPI_GXE_ to SNPs showing high PPLD in the same data set, raising a concern regarding the possibility of higher Type 1 error rates. However, as discussed in Supplemental Material, selecting SNPs based on high PPLD does not appear to increase the rate of false positive findings, which remains low ($\approx$ 1%) for even a lenient threshold of 0.04.

We considered in some detail the question of “outliers,” individuals with high LOA that could be due to their primary DMD mutations rather than any modifier gene effects. We found that late LOA occurred under virtually all of our interaction models, so there is no prima facie reason to exclude such individuals. Indeed, they can drive power to detect interactions, insofar as dropping high LOA individuals can cause power to detect true interactions to plummet. But at the same time, including “phenocopies” has very much the same effect: failing to drop them can also cause power to plummet. We have been as conservative as possible in excluding from our data set individuals with primary *DMD* mutations that might be allowing some residual dystrophin production, but we cannot be certain to have removed all such mutations. We conclude that when a new class of mutations is called into question as potentially allowing some dystrophin production, as happened with exon 2 duplications, we need to analyze the data both including and excluding these individuals in order to avoid missing true effects. We note as well that in the case of Dup2, we have postulated that there may be a post-transcriptional steroid-mediated ameliorative effect due to increased utilization of an exon 5 internal ribosome entry site (IRES) allowing some low-level dystrophin expression, as we have demonstrated that the activity of the dystrophin IRES is increased by corticosteroid exposure [19].

One notable feature of our simulations is the very large degree of sampling variability in results from one replicate to another, and within replicates when we randomly dropped relatively small numbers of individuals. This means that even at a true modifier locus involving interaction with steroid exposure, results can vary widely from one data set to another. Our simulations do not strike us as overly complex, but they do entail several sources of variability: variable allele frequencies and randomized genotypes; randomly generated age at LOA and age at observation (determining censoring status); and random assignment to a covariate class. All of these sources of variation will apply to real data as well in the presence of genetic effects, which means that we can expect random variation among different datasets to result in substantial variations in results. This is particularly true for small to moderate sample sizes. Because this is a point about the data themselves, it applies equally to alternative approaches to data analysis. Indeed, high levels of variability are indicative of all of our generating models. This complicates attempts at independent replication, which is not to say that statistical results do not require validation, but that we should perhaps expect this validation to come from bioinformatic and biological assays, and not necessarily from statistical findings in other datasets.

One limitation of the PPI_G×E_ is that it cannot be effectively applied on a genome-wide basis at our current sample size. Under the null hypothesis of “no effect” (that is, allowing for a steroid effect but no genotypic effect and therefore no interaction), and based on 1 M replicates, we found that 0.62% of simulation replicates returned PPI_G×E_ $\ge$ 0.40; by contrast, the association statistic (TE-PPLD) used in [11] returned a score $\ge$ 0.40 for just 1/1,000,000 simulated SNPs. This discrepancy reflects the fact that there is less information in these data regarding interactions than there is regarding primary associations, hence a noisier null distribution. At our current sample size, therefore, if we were to apply PPI_G×E_ genome wide to 1,000,000 SNPs, we could expect to see 6200 large signals, virtually all of which would (presumably) be Type 1 errors. In selectively following up on the short list of 12 SNPs showing primary association evidence, we would expect just 0.07 (that is, virtually no) false positive results. Since as the sample size increases the PPI_G×E_ will tend to become larger under the alternative hypothesis and smaller under the null, its application on a genome-wide basis could become effective in larger sample sizes.

A striking feature of our results is that we find compelling evidence of genotype x steroid interaction in pathways regulating TGF-β bioavailability. It is perhaps not surprising that the effects of steroids depend on genotypes at *LTBP4* and *THBS1*, consistent with the longstanding hypothesis corticosteroids may blunt inflammation and secondarily mitigate the fibrosis-driving effects of TGF-β in DMD. We also find evidence of dependency of steroid effects on genotype at *CD40* after excluding Dup2 patients; because glucocorticoids can activate the *DMD* exon 5 IRES, the Dup2 subgroup can confound analyses of steroid response. Mechanistically, macrophage activation through *CD40* elicits a pro-inflammatory NF-κB response [9] that operates in parallel, and intersects, the TGF-β pathway. The final gene for which we find evidence of steroid interaction is *NCALD*, which is not explained by involvement in the TGF-β pathway. We note, however, that the association is not with the *NCALD* gene itself but with the rs72681143 SNP in proximity to it. The same region shows multiple *cis*-expression quantitative trait loci (eQTL) for other genes, including *KLF10*, a zinc-finger Krüppel-like transcription factor activated early in TGF-β signaling and whose absence in the *mdx* model exacerbates fibrosis [20]. In total, our novel analysis provides confirmatory evidence that genotypic variation within genes in the TGF-β pathway contributes to overall variability in DMD progression at least in part by modifying the efficacy of steroid administration, further corroborating our understanding of the role of genes in this pathway as DMD modifiers. Equally interesting is the fact that we found evidence *against* steroid interaction for the 5 remaining genes with evidence of a primary association with LOA in our cohort, consistent with the hypothesis of different mechanisms of action for these genes as discussed in [11].

## Supplementary information

Supplemental Material

## Data Availability

The data analysed during the current study are available in the dbGaP repository at https://dbgap.ncbi.nlm.nih.gov/beta/study/phs003680.v1.p1/#study.
