# Supplementary material for: Gene x environment interaction analysis confirms genetic modifier effects on steroid efficacy via TGF-β pathway in Duchenne muscular dystrophy
Source: Eur J Hum Genet. 2026 Apr 20;34(7):964–71. doi: 10.1038/s41431-026-02110-0 (PMC13342305; doi:10.1038/s41431-026-02110-0)
Supplement: Supplementary file 1 — Supplemental Material [file 41431_2026_2110_MOESM1_ESM.pdf]

Supplemental Material

In this section we (A) provide details of the models and methods used for the simulations, and (B) give further details on the statistical methods and implementation of the $PPI_{G\times E}$. In (C) we include some additional results and discussion.

A. Simulation Models and Methods

1. Simulation Generating Models

Supp Table 1 shows the generating models used for the simulations. The first two models are "null" (N) models: N1 is a "Steroid Only" model, with no genotypic effect and therefore no interaction; and N2 is a "No Interaction" model, with both steroid and genotypic effects, but no steroid x genotype interaction. The remainder of the generating models are "alternative" (A) models, which include interaction effects. These and are named under the following conventions: mode of inheritance (Add (additive), Dom (dominant), Rec (recessive) or Oth (other, e.g., over-dominant)) in the steroid N group (first) and the steroid Y group (second), with effects on means only indicated by "M," effects on standard deviations only indicated by "S," and effects on both indicated by "MS." Models labeled "Deleterious" are those in which the rare allele lowers mean LOA. Multiple models with the same modes of inheritance and types of effects are sequentially numbered, moving from smaller effect sizes to larger ones. For instance, AddAddM3 would be the third model with additive inheritance in both the steroid negative and positive groups, with effects on means only. Note that in the simulations, the baseline steroid effect (+ 3 years average LOA; 1.5 x standard deviation) is superimposed on top of the effects as shown in Table 2 for all models.

Supp Table 1 Simulation generating models for genotypic effects on LOA

Model	Description	$\mu_{11,N}$	$\mu_{12,N}$	$\mu_{22,N}$	α_{11}	α_{12}	α_{22}	$\sigma_{11,N}$	$\sigma_{12,N}$	$\sigma_{22,N}$	β_{11}	β_{12}	β_{22}
**N1**	**Steroid Only**	9.1	9.1	9.1	0	0	0	3.3	3.3	3.3	1	1	1
**N2**	**No Interaction**	10.5	9.5	8.5	0	0	0	3.4	3.4	3.4	1	1	1
**A1**	**AddAddM1**	11	10	9	4	2	0	2	2	2	1	1	1
**A2**	**AddAddM2**	11	10	9	5	2.5	0	2	2	2	1	1	1
**A3**	**AddAddM3**	12	10	8	4	2	0	2	2	2	1	1	1
**A4**	**AddAddM4**	12	10	8	5	2.5	0	2	2	2	1	1	1
**A5**	**AddAddS1**	11	10	9	0	0	0	2	2	2	3	1.5	1
**A6**	**AddAddS2**	11	10	9	0	0	0	2	2	2	4	2	1
**A7**	**AddAddS3**	12	10	8	0	0	0	2	2	2	5	2.5	1
**A8**	**AddAddMS1**	11	10	9	2	1	0	2	2	2	3	1.5	1
**A9**	**AddAddMS2**	11	10	9	4	2	0	2	2	2	1.5	1.25	1
**A10**	**AddAddMS3**	11	10	9	4	2	0	2	2	2	3	1.5	1
**A11**	**AddAddMS4**	12	10	8	4	2	0	2	2	2	3	1.5	1
**A12**	**RecRecMS1**	11	9	9	2	0	0	2	2	2	3	1	1
**A13**	**RecRecMS2**	11	9	9	4	0	0	2	2	2	3	1	1
**A14**	**DomDomMS1**	11	11	9	2	2	0	2	2	2	3	3	1
**A15**	**DomDomMS2**	11	11	9	4	4	0	2	2	2	3	3	1
**A16**	**RecOthS**	11	9	9	0	0	0	2	2	2	1	3	1
**A17**	**DomOthS**	11	11	9	0	0	0	2	2	2	1	3	1
**A18**	**RecOthMS**	11	9	9	4	0	0	2	2	2	1	3	1
**A19**	**DomOthMS1**	11	11	9	4	4	0	2	2	2	1	3	1
**A20**	**DomOthMS2**	11	11	9	4	4	0	2	2	2	1	4	1
**A21**	**DeleteriousM1**	9	10	11	-1	-0.5	0	2	2	2	1	1	1
**A22**	**DeleteriousM2**	9	10	11	-2	-1	0	2	2	2	1	1	1
**A23**	**DeleteriousM3**	9	10	11	-3	-1.5	0	2	2	2	1	1	1

Models are shown in ages, with means (μ's) and standard deviations (σ's) shown for the steroid N group, and changes to means (α's, representing additive changes to μ's) and changes to standard deviations (β's, representing multiplicative changes to σ's) for the steroid Y group, for each genotype (11, 12, 22), respectively. In addition to effects represented in the table, steroid exposure adds 3 years to LOA for all genotypes and a factor of 1.5 standard deviations for all genotypes, for all simulating models including N1, N2. Note that some generating models might give rise to negative ages; this happens particularly when the mean age is low and the standard deviation is high. For this reason, in simulating the data, these parameter values define left-truncated (at 0) normal distributions.

As an aid to understanding these models, Supp Figure 1 illustrates the generating conditions for one particular model (A14), showing the LOA distributions from which the simulated data are drawn, first without steroid effects (so that the numbers in the figure correspond exactly to what is shown in Supp Table 1), and then with steroid effects included (as described in the footnote to Supp Table 1).

Supp Fig 1 Illustration of simulated LOA distributions by genotype (11, 12, 22) and Steroid group.

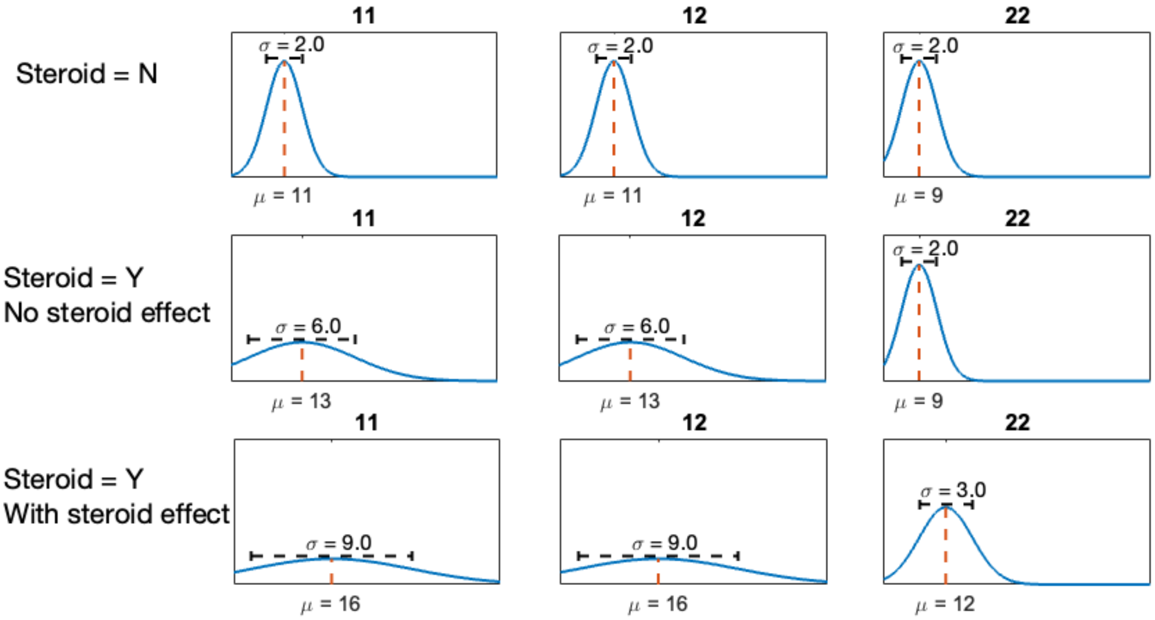

2. Simulation Generating Methods

For each generating model, an individual was simulated based on a random draw of $t_e(x_i)$ from an age-at-event (AE) distribution, as given by the generating parameters (see above) and incorporating left-truncation of the distribution to avoid negative ages, and an independent random draw of $t_o(x_i)$ from an age-at-observation (AO) distribution. AO was simulated under a negative binomial distribution with $r = 11$, $p = 0.4$. If $t_e(x_i) < t_o(x_i)$, the individual was considered uncensored with failure time $t_{fail}(x_i) = t_e(x_i)$; otherwise, the individual was considered censored with censoring time $t_{cens}(x_i) = t_o(x_i)$. This procedure yields a censoring rate ≈ 25% (depending on the generating model), which is similar to what we have in the real DMD data set (26.7%). LOA is virtually never less than 6 years of age for Duchenne patients, but for some of our simulations small numbers of individuals with LOA < 6 were generated; to better mimic the LOA distribution in real data we simply dropped and replaced any such individuals.

B. Statistical Methods and Implementation Details

1. Residual calculations for analysis of LOA

Following methods detailed in [1, 2] and previously used in [3], LOA was transformed onto the Ordinary Time-to-Event (OTE) residual scale as input to PPI_{GxE} analysis. This step involves assigning a predicted LOA for censored individuals, based on the conditional estimated survival function given $t_o(x_i)$. Throughout the main text, we use "LOA" to refer to ages on the original

scale, utilizing observed LOA for uncensored individuals and predicted LOA for censored individuals.

In previous applications we fit survival functions separately in the Steroid Y and N groups, in order to essentially regress out steroid effects. Because in this application we are interested in modeling the steroid effects themselves, we fit a single survival function for the entire dataset when assigning OTE values.

2. Form of the Likelihood Ratio (LR_{GxE})

Our formulation of the hypotheses gives rise to a non-standard form of likelihood ratio: in the expression $LR(H_1, H_2) = \frac{P(data \mid H_2)}{P(data \mid H_1)}$, all factors corresponding to the steroid N group cancel out, as these are the same under both hypotheses. Thus the parameters referring to this group become unidentifiable. For this reason, we first obtain maximum likelihood estimates of the 8 parameters corresponding to H_1 ($\mu_{11,N}, \mu_{12,N}, \mu_{22,N}, \sigma_{11,N}, \sigma_{12,N}, \sigma_{22,n}, \alpha, \beta$) from the data, and insert these values into the LR This leaves an LR with 6 free and identifiable parameters ($\alpha_{11}, \alpha_{12}, \alpha_{22}, \beta_{11}, \beta_{12}, \beta_{22}$).

3. Handling related individuals

There are some related individuals in the UDP dataset (17 families with 36 total individuals). Only 1 (randomly selected) individual from any given family is included in the procedures used to estimate the survival function in computing OTE's. But all relatives are included in the $BR_{G\times E}$ calculations, under the assumption that conditional on steroid exposure and SNP genotype, LOA among relatives is uncorrelated. While this assumption may not hold exactly, it only affects cases in which the relatives share both steroid status and genotype, so that numerical effects on $BR_{G\times E}$ for any given SNP are minimal.

4. Setting a minimum standard deviation in calculating the "null" likelihood

When the MAF is low, there may be very few observations in any given cell, particularly for individuals with the 11 (rare) genotype in the smaller Steroid Negative group. In this case, it is possible to have artificially low estimated standard deviations. Recall that the $BR_{G\times E}$ calculation is based on (1) estimating the parameters of the "null" distribution and fixing these parameters in the denominator of the LR, and only then (2) integrating the LR over the parameter space. In cases in which the estimate of the standard deviation (for a given cell) is extremely low, the resulting "null" likelihood can be highly deflated, leading to highly inflated $BR_{G\times E}$ under the null hypothesis. For this reason we set a minimum standard deviation (minSTD) for all cells when calculating these estimates. Supp Fig2 shows results under the "No Interaction" model. As can be seen, the median $BR_{G\times E}$ starts increasing as the minSTD drops below 0.5. This illustrates why setting this minSTD is protective against spurious "false positives."

Supp Fig 2 (a) Boxplots of $BR_{G\times E}$ as a function of the minimum estimated standard deviation (minSTD), with minSTD grouped in bins of size of 0.05; (b) detail of the y-axis from plot (a).

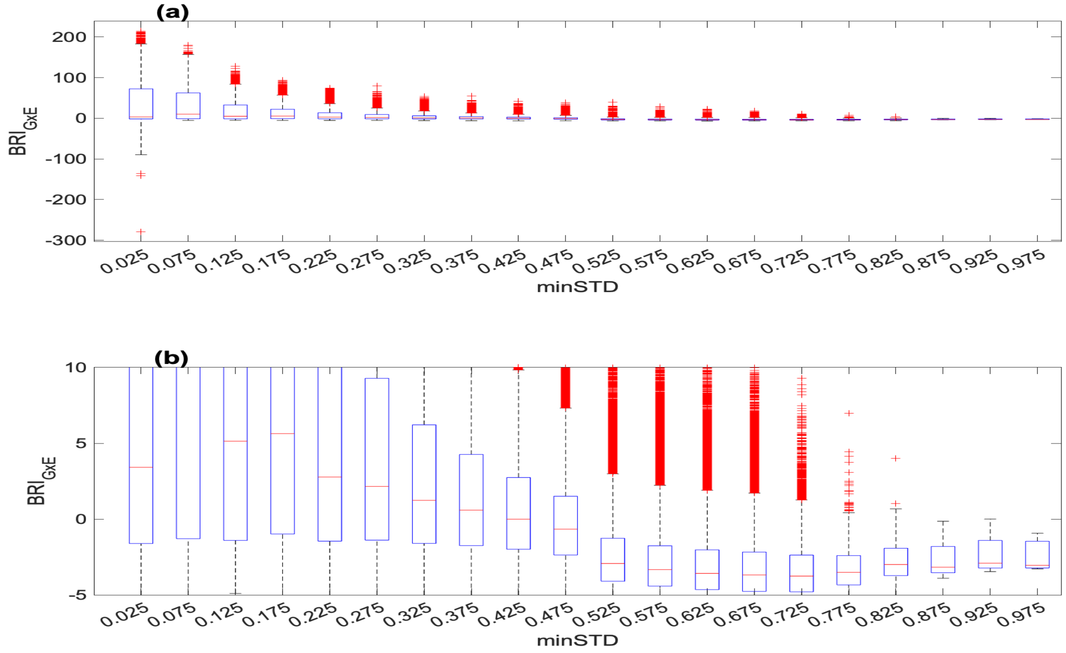

5. Dropping cells with low counts

Even after setting minSTD = 0.50, we find that in cases with very low cell counts, the $BR_{G\times E}$ can be spuriously large under the null hypothesis, as shown in Supp Fig 3.

Supp Fig 3 Boxplot for $BR_{G\times E}$ as a function of the minimum cell count for genotype 11, after setting minSTD = 0.50.

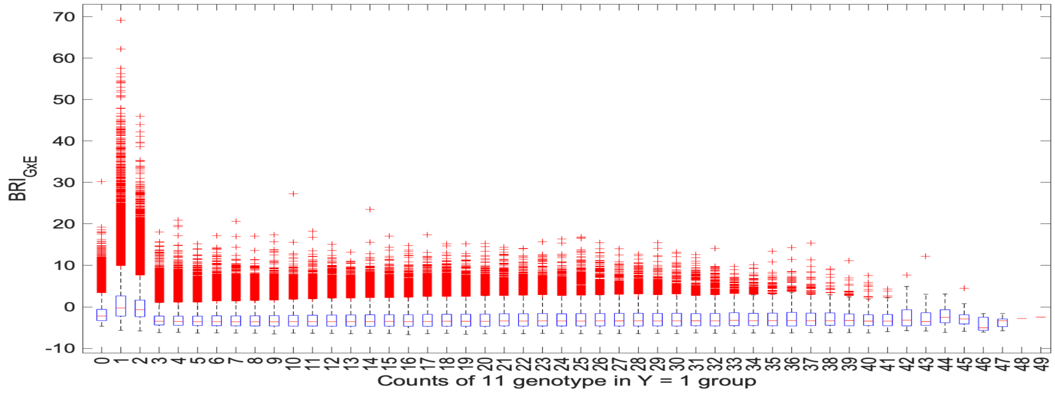

For this reason, if the number of individuals in any steroid x genotype cell is < 3, we simply drop that genotype from the LR calculation (or equivalently, we set the LR for that cell to 1).

6. Integration over parameter space

The reason for integrating over the parameter space, rather than simply using the maximum $BR_{G\times E}$ (or "MOD") as the basis for our test statistic is that the MOD can never indicate evidence in favor of the null, only against it. But a key feature of our statistical methodology is accumulation of evidence either for or against interaction, such that as the sample size increases the distributions of the statistic between "evidence for" and "evidence against" are increasingly non-overlapping. Integration achieves this goal, but it introduces other numerical issues.

In keeping with our previous work, as referenced in the main text, we use a uniform prior for the integration; equivalently, we take a straightforward *average* of LRs over the parameter space. To do so, we need to impose limits on the range of integration, since LOA is a continuous trait. Our procedure is as follows. (1) We standardize LOA, centering the data at 0. This allows us to implement a single procedure for all calculations, rather than having to handle each model individually. (2) We then center the range of integration over the maximum likelihood estimates for each of the 6 parameters. This ensures that we always capture the maximum likelihood model. (3) We then integrate, for each genotype i, over α_i within 2 standard deviation of its m.l.e., and over a multiplicative range of 1/2 to 2 times each β_i. This is our "standard range."

In general, $BR_{G\times E}$ is a simple linear function of the MOD, with the slope of the function depending in a regular way on the width of the integration interval. Clearly as we narrow the ranges of integration, the $BR_{G\times E}$ will approach the MOD; conversely, the broader the ranges of integration, the smaller the $BR_{G\times E}$ will become. Again, our primary purpose in integrating the LR at all is to be able to obtain evidence against (as well as in favor of) interaction, but the size of the $BR_{G\times E}$ depends on the range of integration. Our primary concern is therefore to avoid situations in which we might erroneously infer evidence either for or against interaction due solely to over-restriction of the range.

In selecting our standard range we tried various options and compared their behavior. Here we show results for a "wide range," in which we integrate over each α_i within 3 standard deviation of its m.l.e., and over a multiplicative range of 1/2 to 3 times for each β_i. The first thing to note (Supp Fig3) is that the difference $\Delta = BR_{G\times E}(\text{standard}) - BR_{G\times E}(\text{wide})$, is constant as a function of $BR_{G\times E}(\text{standard})$. This is a general result and not specific to any one generating model, as illustrated in Supp Fig 4. The second thing to note is that the mean (standard deviation) of Δ is 0.81 (0.14). Note that our heuristic thresholds for weak, moderate and strong evidence (PPI_{GxE} = 0.04, 0.10, 0.40) correspond to $BR_{G\times E}$ = 2.02, 2.44 and 3.22, respectively. This means that increasing the integration width is very unlikely to convert positive results to negative ones, even for the lowest threshold. And of course scores that are negative using the standard width cannot be increased by widening the integration range. We conclude that integration does permit us to distinguish evidence against interaction from evidence for interaction, and that our standard range is sufficient to accomplish this aim without danger of misleading us, particularly for negative $BR_{G\times E}$ or for positive $BR_{G\times E}$ outside the indeterminate range (say, in our application, $BR_{G\times E} \geq 3$).

Supp Fig 4 Scatter plot of $\Delta = BR_{G\times E}(\text{standard}) - BR_{G\times E}(\text{wide})$ as a function of $BR_{G\times E}(\text{standard})$ for models (a) AddAddMS1 and (b) RecOthMS1

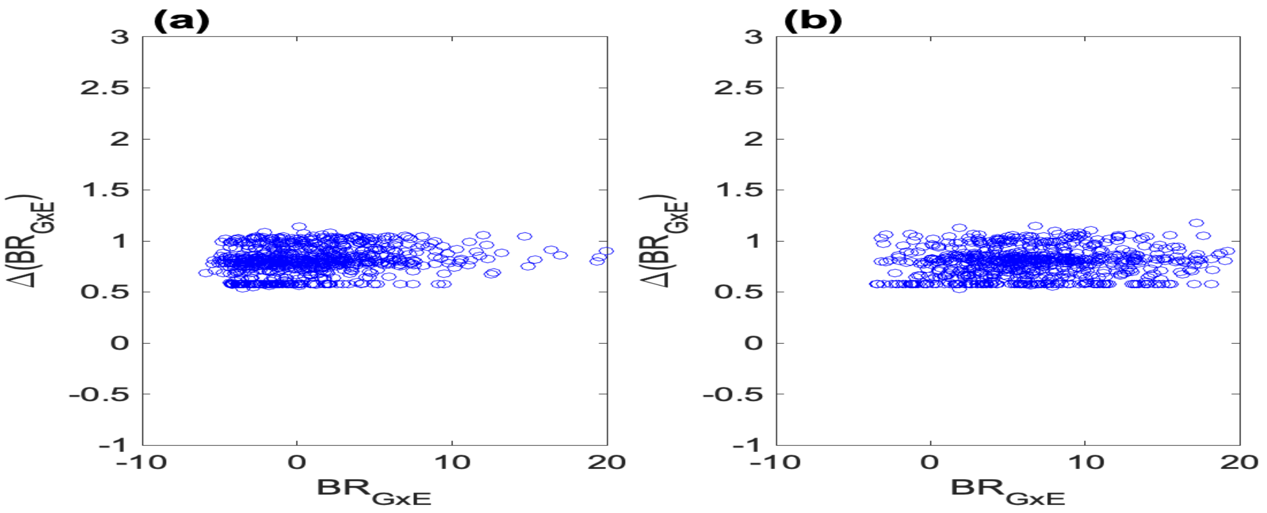

C. Supplemental Results

1. Effects of point-prior on results

As discussed in the main text, the use of a prior probability $\boldsymbol{\pi}$ of interaction = 0.0004 is based on scaling considerations, and is somewhat arbitrary. However, as shown here, reducing $\boldsymbol{\pi}$ by even 3 orders of magnitude has virtually no effect on our main results. The one exception is CD40, for which reducing $\boldsymbol{\pi}$ by 1-2 orders of magnitude does notably affect the PPI_{GxE}.

Supp Table 2

	**PPI_{GxE}**			
**$\boldsymbol{\pi}$**	**LTBP4 (N=419)**	**THBS1 (N=419)**	**NCALD (N=407)**	**CD40 (N407)**
0.0004	1.0000	0.9999	1.0000	0.5403
0.00004	1.0000	0.9995	1.0000	0.1052
0.000004	1.0000	0.9950	1.0000	0.0116
0.0000004	0.9998	0.9521	1.0000	0.0012

2. *Effects of 2-stage design on Type 1 error rates*

We have utilized a two-stage study design, in which we first selected SNPs based on large PPLD, and then tested those selected SNPs for steroid interaction based on the same dataset. One question that arises is whether using the same data set for both stages might be increasing the false positive rate. We note first that the PPLD itself is conservative: as reported in [3]: in 1 million replicates simulated under “no trait-marker association,” we observed only 7 PPLDs ≥ 0.10. Thus the chances that any one of our selected SNPs represents a false-positive in the sense of having no actual effect on LOA is low. However, the selection procedure might inflate the distribution of PPI_{GxE} under the hypothesis that there is a primary association but no steroid interaction. To assess this, we modified our N2 model in order to obtain a large proportion of primary PPLDs ≥ 0.10, and then split the 1,000 replicates into two groups: those with PPLD ≥

0.10 and those with PPLD < 0.10. We then calculated PPI_{GxE} for each replicate. Specifically, this new model has LOA means in the Steroid = N group of 11, 9.5, 8.0 for genotypes 11, 12, 22, respectively (association effect on the means); and standard deviations of 2.0 for all genotypes. For each genotype, $\alpha = 0$ and $\beta = 1$ (no steroid interaction). Out of 1,000 replicates, 581 had PPLD ≥ 0.10, and the average PPLD was 0.47. Supp Table 3 shows the proportion of replicates with PPI_{GxE} crossing various thresholds.

Supp Table 3

Proportion of replicates crossing threshold under "association but no interaction"

	**PPI_{GXE} Threshold**			
	**< 0.0004**	**≥ 0.04**	**≥ 0.1**	**≥ 0.4**
PPLD ≥ 0.10	95.87	1.20	0.86	0.86
PPLD < 0.10	95.70	1.19	1.19	0.72

As can be seen, false-positive rates remain low, hovering just under 1%, in both groups even for the 0.04 threshold, while true-positive rates are ≈ 96% in both groups. Based on only 1,000 replicates it is hard to precisely quantify the rate of high outlier values; the exact rates will also depend on the association generating model. Supp Fig 5 shows the distribution of PPI_{GxE} as a function of PPLD across the 1,000 replicates. Note that the distribution of PPLD itself is sparser in the center of the PPLD range; the false-positive rates are probably not actually higher at both ends of the PPLD range. The correlation coefficient for this model is -0.00.

Supp Fig 5 Scatter plot of PPIGxE as a function of PPLD under the null model of "association but no interaction."

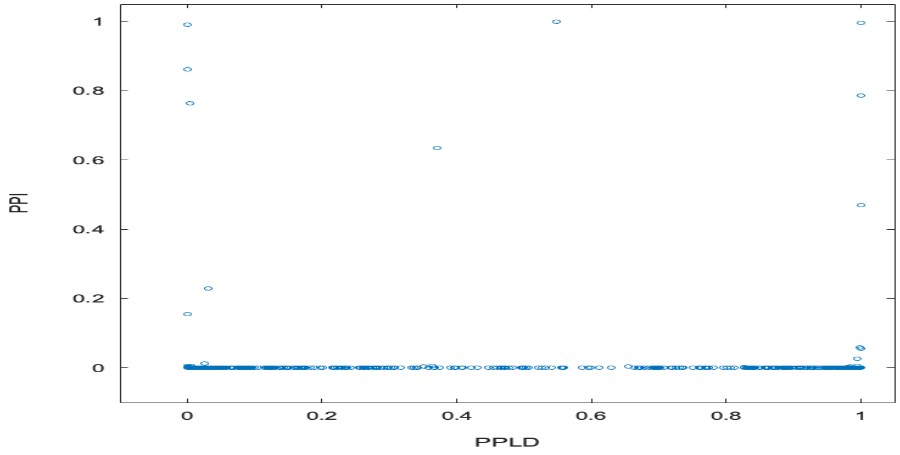

3. *Estimated trait models for 4 salient PPI_{GxE} findings*

Supp Tables 4 and 5 show maximum likelihood estimates of the trait parameters for the 4 SNPs with high PPI_{GxE}.

Supp Table 4 Estimated mean $\hat{\mu}$ and standard deviation $\hat{\sigma}$ by genotype, in the steroid negative (N) and steroid positive (Y) groups respectively, for each of our 4 salient PPI_{GxE} findings.

	**Genotype**					
	**11**		**12**		**22**	
	$\hat{\mu}$	$\hat{\sigma}$	$\hat{\mu}$	$\hat{\sigma}$	$\hat{\mu}$	$\hat{\sigma}$
LTPB4						
**N**	12.21	3.55	10.40	2.00	11.11	3.42
**Y**	13.39	3.65	12.49	3.13	12.47	2.84
THBS1						
**N**	13.10	0.63	11.13	1.99	10.78	3.35
**Y**	13.63	1.70	12.65	3.23	12.49	2.97
NCALD						
**N**	---	---	9.48	1.86	10.96	2.52
**Y**	---	---	12.15	4.24	12.49	2.46
CD40						
**N**	10.63	2.63	10.65	2.66	10.69	2.33
**Y**	13.02	3.22	11.83	2.38	12.80	3.20

Note: For NCALD, there is only 1 individual with genoptype 11 in the Steroid N group, and none in the Steroid Y group. This genotype is therefore not included in the analyses for this SNP.

Supp Table 5 Estimated overall $\hat{\alpha}, \hat{\beta}$ and genotype-specific $\hat{\alpha}, \hat{\beta}$ for these same genes (obtained from table above).

	$\hat{\alpha}$	$\hat{\beta}$	$\hat{\alpha}_{11}$	$\hat{\alpha}_{12}$	$\hat{\alpha}_{22}$	$\hat{\beta}_{11}$	$\hat{\beta}_{12}$	$\hat{\beta}_{22}$
LTBP4	1.62	1.00	1.18	2.09	1.38	1.03	1.57	0.83
THBS1	1.62	1.00	0.53	1.52	1.70	2.71	1.62	0.89
NCALD	1.75	1.18	---	2.68	1.53	---	2.28	0.98
CD40	1.75	1.18	2.38	1.19	2.10	1.22	0.90	1.37

References

1. Vieland VJ, Seok S-C, Stewart WCL. A new linear regression-like residual for survival analysis, with application to genome wide association studies of time-to-event data. PLoS One. 2020;15(5):e0232300.
2. Vieland VJ, Seok SC. The PPLD has advantages over conventional regression methods in application to moderately sized genome-wide association studies. PLoS One. 2021;16(9):e0257164.
3. Flanigan KM, Waldrop MA, Martin PT, Alles R, Dunn DM, Alfano LN, et al. A genome-wide association analysis of loss of ambulation in dystrophinopathy patients suggests multiple candidate modifiers of disease severity. Eur J Hum Genet. 2023;31(6):663-73.
